# 3D surgical instrument collection for computer vision and extended reality

**DOI:** 10.1038/s41597-023-02684-0

**Published:** 2023-11-11

**Authors:** Gijs Luijten, Christina Gsaxner, Jianning Li, Antonio Pepe, Narmada Ambigapathy, Moon Kim, Xiaojun Chen, Jens Kleesiek, Frank Hölzle, Behrus Puladi, Jan Egger

**Affiliations:** 1 Institute of Computer Graphics and Vision (ICG), Graz University of Technology, Inffeldgasse 16/II, 8010 Graz, Austria; 2 Institute for Artificial Intelligence in Medicine (IKIM), Essen University Hospital (AöR), Girardetstraße 2, 45131 Essen, Germany; 3 Institute of Biomedical Manufacturing and Life Quality Engineering, State Key Laboratory of Mechanical System and Vibration, School of Mechanical Engineering, Shanghai Jiao Tong University, 800 Dongchuan Road, 200240 Shanghai, People’s Republic of China; 4 Cancer Research Center Cologne Essen (CCCE), West German Cancer Center Essen (WTZ), 45122, Essen, Germany; 5 Technische Universität Dortmund, Fakultät Physik, Otto-Hahn-Straße 4, 44227 Dortmund, Germany; 6 Department of Oral and Maxillofacial Surgery, University Hospital RWTH Aachen, Pauwelsstraße 30, 52074 Aachen, Germany; 7 Institute of Medical Informatics, University Hospital RWTH Aachen, Pauwelsstraße 30, 52074 Aachen, Germany; 8 Center for Virtual and Extended Reality in Medicine (ZvRM), University Hospital Essen, Hufelandstraße 55, North Rhine-Westphalia 45147 Essen, Germany

**Keywords:** Databases, Engineering

## Abstract

The availability of computational hardware and developments in (medical) machine learning (MML) increases medical mixed realities’ (MMR) clinical usability. Medical instruments have played a vital role in surgery for ages. To further accelerate the implementation of MML and MMR, three-dimensional (3D) datasets of instruments should be publicly available. The proposed data collection consists of 103, 3D-scanned medical instruments from the clinical routine, scanned with structured light scanners. The collection consists, for example, of instruments, like retractors, forceps, and clamps. The collection can be augmented by generating likewise models using 3D software, resulting in an inflated dataset for analysis. The collection can be used for general instrument detection and tracking in operating room settings, or a freeform marker-less instrument registration for tool tracking in augmented reality. Furthermore, for medical simulation or training scenarios in virtual reality and medical diminishing reality in mixed reality. We hope to ease research in the field of MMR and MML, but also to motivate the release of a wider variety of needed surgical instrument datasets.

## Background and Summary

Instruments for surgical procedures have been found in archaeological excavations from the bronze age and as paintings on stone, or paintings on walls of tombs and on papyrus^1^. The various instruments such as forceps, hooks or retractors have undergone a noticeable evolution, especially since the 18th century^2^. Due to the continuing specialisation in surgery over the last decades, a wide variety of specialised surgical instruments have been developed^1^. However, there is still a common pool of instruments such as scissors, scalpels, retractors, forceps and the like^2^. As an interventional discipline like surgery, dentistry has a distinct set of special instruments for the treatment of dental diseases^3–6^. Furthermore, there are now iso norms in the property and nature of surgical instruments to meet the special requirements in the human situs (ISO 7151:1988, ISO 7153-1:2016, ISO 7740:1985, ISO 7741:1986, ISO 13402:1995, ISO 6360-3:2005).

Recent developments in deep learning enable advanced computer-assisted surgery systems, by detecting and differentiating surgical tools, tracking their movements, and providing feedback to the surgeon^7^. However, surgical data science relies on large-scale data sets of tools and their movement. Current datasets contain images and videos of surgical scenes, including bounding boxes, labels or segmentation masks^8,9^. This limits their utilisation to very specific applications^10,11^.

Synthetic datasets have become a necessity in the computer vision domain^12,13^. Furthermore, synthetic data has shown to be a potentially valuable addition to training machine learning models^14^. As the generalisation ability of deep learning models increases, synthetic datasets are becoming an enticing alternative or addition for training them^13–19^.

In this context, a dataset of three-dimensional (3D) medical instruments can be used to create realistic surgical scenes, applicable for training deep algorithms for instrument detection, segmentation or marker-less 3D instrument registration and tracking^20^. Aside from surgical data science, 3D models of medical instruments have applications in medical simulation or training scenarios in virtual reality^21^ and medical mixed reality^22,23^. They can aid in simulating and planning the instrument path in surgeries virtually. Virtual surgery planning already occurs frequently in oral and maxillofacial surgery^24–26^.

Therefore, we present a collection of 3D models of a wide variety of instruments from surgery and dentistry. In contrast to existing datasets, our collection can be used to generate and render almost unlimited realistic scenes, both 2- and 3- dimensional.

In this paper, we describe our unique data collection, which contains scanned 3D models of 103 medical instruments. The instruments within our collection are mainly related to dentistry and oral and maxillofacial surgery but are not limited to them.

The 3D meshes of the surgical instruments were virtually replicated by scanning instruments with structured light scanners and by subsequent post-processing performed in the proprietary scanning software. All models were visually inspected, analysed and classified into groups such as retractors, forceps and clamps.

We also demonstrate a method to increase the total number of models in the collection. The approach effectively creates new variations of the original model by semi-manually adjusting, scaling and smoothing the original. This can be seen as a form of data augmentation for generating massive datasets, which can be beneficial for deep learning^13,27–29^.

## Methods

**Fig. 1:**
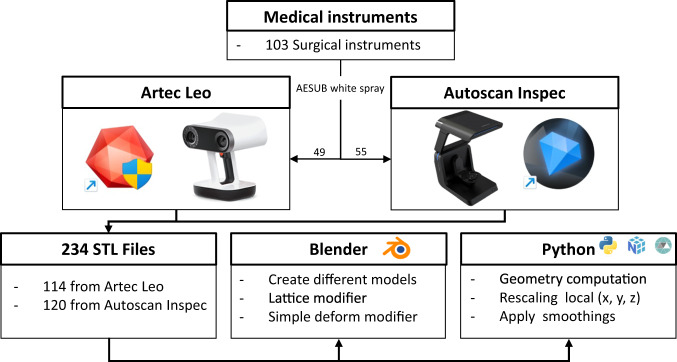
Flowchart for generating the 3D Models within this collection. An estimation based on the specifications of the scanner (Artec Leo or Autoscan Inspec) was made to choose a scanner per instrument. If results were deemed insufficient, an attempt was made with the other scanner. We furthermore provide a Blender add-on and Python script to create a multitude of likewise models (e.g., via deformation, rotating or translating a part of the mesh, scaling and smoothing), which allow the generation of an even larger-scale dataset of geometrically plausible instruments. The original STL files are analysed using Python, utilising the Numpy and Trimesh libraries.

The data collection consists of four key steps: instrument preparation for scanning purposes, 3D scanning using structured light scanners, post-processing in proprietary software, analysis of the generated models, and model adaptation to create a variety of models based on the originals. Figure 1 provides an overview of our pipeline, which we will now describe in detail.

103 surgical instruments were used to create the dataset. These instruments were kindly provided by the Department of Oral and Maxillofacial Surgery of the University Hospital Rheinische-Westfälische Technische Hochschule (RWTH) Aachen. The study was conducted at the Institute of Artificial Intelligence in Medicine (IKIM) of the Essen University Hospital (AöR). For scanning, the instruments were prepared with AESUB white (Scanningspray Vertriebs GMBH, Recklinghausen, North Rhine-Westphalia, Germany).

Two structured light 3D scanners, the Autoscan Inspec (Shining 3D Corporation, Hangzhou, Zhejiang, China) and the Artec Leo (Artec3D, Senningerberg, Canton Luxembourg, Luxembourg), were used for obtaining the 3D models. Post-processing was done with their respective commercially available proprietary software UltraScan version 2.0.0.7 and Artec Studio 17 Professional version 1.0.141. All models were exported from the proprietary software as Stereolithography (STL) files (Stereolithography Interface Specification, June 1988, 3D Systems Inc., Valencia, California, United States).

Additional visual inspection was done in Microsoft 3D Viewer (Microsoft Corporation, Redmond, Washington, United States) and Blender 3.4.1 (The Blender Foundation, Amsterdam, Noord-Holland, Netherlands https://blender.org). A Blender add-on based on Python was implemented, to alter original models and, thus, enabling the creation of a plethora of likewise models based on the original scanned instrument. Another method of creating likewise models can be fully automatically achieved using a Python 3.9 (The Python Software Foundation, Wilmington, Delaware, United States https://python.org) script. It allows scaling the instruments along the local axes of the original model, or the application of different types of smoothing (Taubin, Laplacian and Humphrey algorithms).

All processing scripts and the Blender add-on are included in the data repository^30^. The original STL models were analysed according to their geometric properties, such as length, width, height and volume. These descriptors, together with all created STL files are available in the data repository^30^. The STL models will also be made available on MedShapeNet (https://medshapenet.ikim.nrw)^31^. Figure 1 shows an overview of all steps involved in producing the data collection. Out of the 103 surgical instruments, 49 underwent scanning with the Artec Leo and 55 with the Autoscan Inspec. One instrument was scanned using both scanners. 11 instruments were scanned in alternative configurations, such as open and closed stances. Eight instruments were scanned in an open stance using the Artec Leo, and three instruments were scanned with the Autoscan Inspec, totalling four supplementary scans. To account for diverse post-processing options, we generated two STL files per scan, representing different settings. In Chapter “Data acquisition and post-processing with Artec Leo and Artec Studio

Professional 17” and “Data acquisition with Autoscan Inspec and UltraScan”, the differences between these two settings are explained. To confirm reproducibility one instrument was scanned and processed by two different people using the Autoscan Inspec resulting in an additional scan. Thus, a comprehensive sum of 114 STL files resulted from scans using the Artec Leo (49 + 8)*2 = 114. Similarly, scans performed using the Autoscan Inspec resulted in 120 STL files, (55 + 4 + 1)*2 = 120. For details on which instruments were scanned multiple times, we refer to the ‘Overview’ Word file within the data repository^30^.

### Instrument preparation

**Fig. 2:**
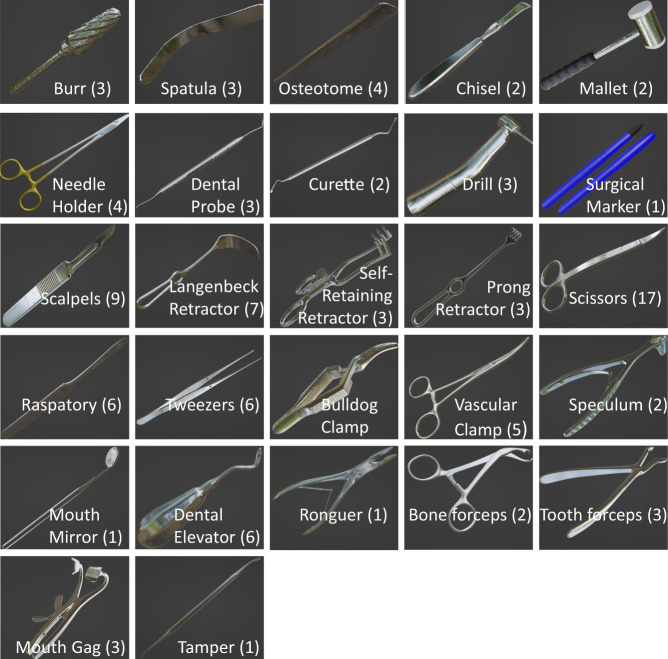
An overview of all 27 classes of instruments included in our collection. The number of examples within each class is listed in parentheses, and one example model per class is shown. Using Blender, each instrument is given one or more materials using only the standard principled shader.

The instruments used in the department of oral and maxillofacial surgery were divided into 27 classes, see Fig. 2. The instruments can be used for a plethora of interventions and actions. For example, retractors provide a clear view, hammers and chisels apply controlled force, clamps hold blood vessels and tissues, forceps grasp and manipulate these tissues, and dental probes examine teeth and gums.

Nearly all instruments are made of stainless steel, due to its durability and resistance to corrosion. The instruments are also smoothly polished. The reflective nature of polished stainless steel causes scatter and limits the accuracy of point cloud data obtained with structured light 3D scanners. Some instruments further have black handles, which absorb the light emitted by the scanner, thus limiting the obtained data severely.

Therefore, to enable appropriate scanned mesh quality, all instruments were prepared with 3D scanning spray. AESUB white, one of the most used 3D scanning sprays, is easily washable but does not evaporate, and has a layer thickness of approximately 0.007 millimetres. These properties are why we deem this spray suitable.

The Artec Leo has a 3D point accuracy of up to 0.1 millimetres and a resolution of up to 0.5 millimetres, a couple of spray layers with AESUB white should not negatively influence the scan resemblance of the real instrument. The Autoscan Inspec however has an Accuracy of 0.01 millimetre and spraying was therefore kept to a necessary minimum.

There are sprays on the market that enable the scanning of an object’s colours or provide a lower layer thickness than AESUB white. For instance, AESUB is transparent, orange, and yellow. However, AESUB orange offers only a slight reduction of a few micrometres in layer thickness, thus still influencing the scan outcome. AESUB yellow necessitates the use of a spray gun and additional expertise in spraying. In our experience AESUB transparent does not substantially decrease the surgical instrument’s reflectivity to allow it to be accurately scanned with the Artec Leo. We concluded that AESUB white is the most suitable choice for scanning our instruments.

**Fig. 3:**
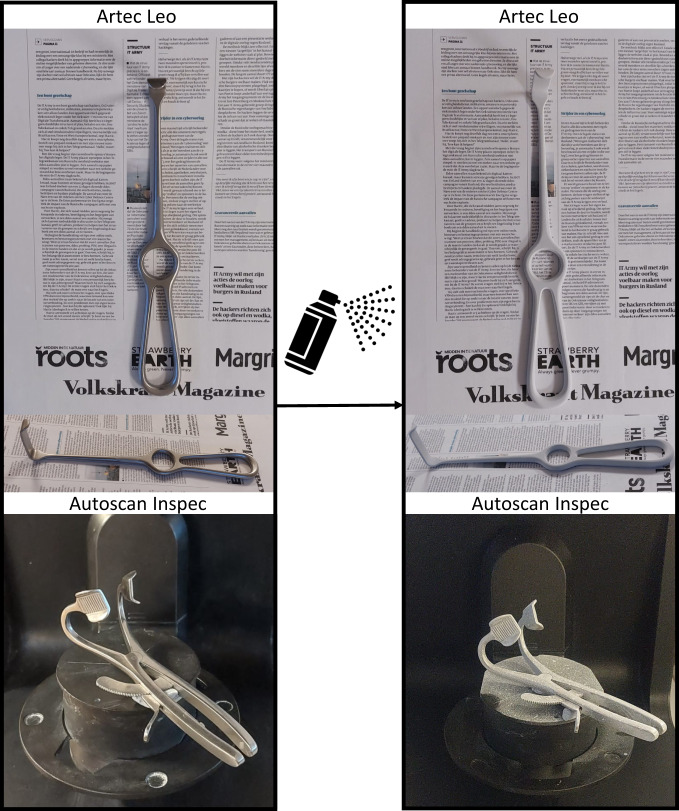
Example for instrument Langenbeck retractor 5 (top) and Mouth gag Denhart 1 (bottom), scanned with Arte Leo and Autoscan Inspec, respectively. The left images show the instruments before the scanning spray has been applied, and the right images show the tools after spraying.

This is especially true since Instruments scanned with the Artec Leo required evenly and fully covering layers of spray. Although the Autoscan Inspec was more adept at handling the reflective, shiny and absorbing properties of our instruments, in our setup, all stainless-steel instruments or parts required a single layer of spray for appropriate scans. In accordance with the manufacturer’s instructions, the scanning spray was always applied by shaking it prior to usage and spraying it at a distance of 15–20 centimetres while slowly and steadily moving around the instrument. An example of the results obtained before and after spraying is shown in Fig. 3. The obvious downside of this method is that the original surface texture cannot be captured, therefore we feel the universal STL format is appropriate for sharing the models created in this study.

### Scanners and post-processing

A desktop computer with an AMD Ryzen 9–5900 × 12 Core Processor and 3.200 hertz DDR4 RAM along with an NVIDIA GeForce RTX 3090 graphics card was used for post-processing, analysing and augmenting the data collection. The 3D point cloud data was obtained using Artec Leo and Autoscan Inspec Structured light scanners. The corresponding software Artec Studio Professional 17 and UltraScan version 2.0.0.7 were used for post-processing and model generation.

### Data acquisition and post-processing with Artec Leo and Artec Studio Professional 17

Artec Leo is a handheld 3D scanner. It utilises a white 12 Light-emitting diode (LED) array light source, with an optimal working distance between 0.35–1.2 metres. An accuracy of 0.2 mm + 0.3 mm/m should be obtainable according to the manufacturer.

As a trade-off between accuracy and the desire to have the whole instrument within our field of view during scanning, 0.5 metre scanning distance is chosen with a recommended exposure time of one millisecond. To guarantee this distance, the scanner was set to show the distance colour map superimposed on the scanned object while scanning. To minimise error, the scanner was set to only scan the object if tracking was maintained. In this context, tracking refers to the automatic estimation of the relative frame position performed by the scanner during recording. Tracking is based on common surface and texture features. Therefore, a simple background rich in texture features was chosen. Recording was performed with 60 high-definition frames per second. Artec Leo allows recording additional texture frames, the combined setting was used to record a total of 65 frames per second. Scanning was done by slowly and smoothly encircling the stationary instrument, changing the angle of the scanner in relation to the instrument throughout all positions in the circle. The actual scanning rate varied due to Artec Leo’s feature of recording only when tracking was accurate. Each scan recorded approximately 800–1200 frames which took less than a minute to acquire.

Each instrument was scanned in two orientations to ensure a complete compassing capture of the instrument for later post-processing. Both scans of the instrument were imported in Artec 17 professional with data density factor 8 for its artificial intelligence-powered enhanced reconstruction. This is a feature from the manufacturer to increase the data points and reduce the noise within the scan. These are also the maximum recommended settings for our hardware setup.

**Fig. 4:**
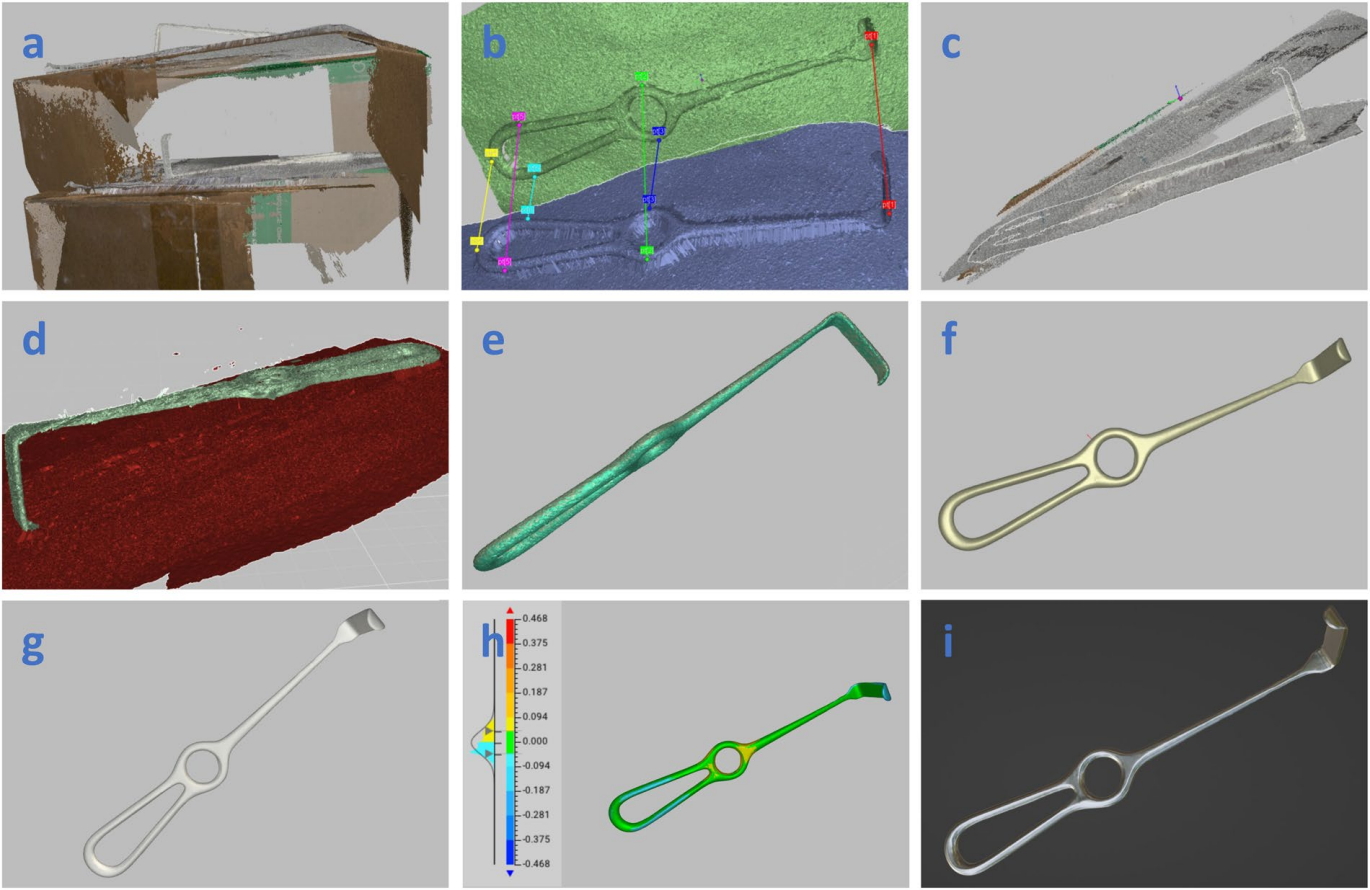
Depiction of the data acquisition and processing workflow for the Artec Leo with the proprietary software, using Langenbeck Retractor 5 as an example. The first step is importing the scans with high-definition data density followed by a global registration (**a**). After cropping, semi-manual alignment is applied (**b**), which results in a coarsely registered scan shown in (**c**), followed by another global registration. The background (**d**, red bottom part) and unwanted artefacts are then removed. (**e**) shows the model after the background and outliers have been removed. (**f**) displays a smooth fusion result, (**g**) shows the smooth fusion with texture (white spray). In (**h**), we confirm that the difference between a smooth and sharp fusion is minimal. (**i**) is an example of how this instrument would look given a realistic metallic look in Blender.

Figure 4 gives an overview of the steps for post-processing objects scanned with the Artec Leo and its proprietary software. After importing the recording and HD reconstruction, the global registration feature was used, and a region of interest was cropped out with the editor tool. The global registration converts all surfaces created from single frames to a single coordinate system. To do so, the software selects geometry and texture points within a frame and matches these points to the other frames. The software then tries to minimize the mean differences between these points. Unfortunately, the exact algorithm is not publicly disclosed by the manufacturer. Frames with an error distance greater than 0.3 mm were not used to generate surface models. To ensure accurate registration, a key frame ratio of 0.5 is used for geometry and texture-based registration, which looks for features within areas of five squared millimetres. Key-frame ratio determines the percentage of frames the software utilises for the registration on a scale of zero to one.

Semi-manual alignment was then performed, Fig. 4b, followed by an additional global registration to compensate for potential earlier mismatches based on features that are now deleted. The background, noise and artefacts were manually removed from both scans using the editor tool. Visual inspection was conducted, and if necessary, additional semi-manual alignment was performed. This was needed in cases where due to a lack of features on the surgical instrument, in combination with its symmetrical shape, the algorithm of the Artec Studio software prioritized background features over instrument features. Subsequently, outlier removal was performed with a 3D-noise level set to three, and accuracy set to 0.2 millimetres, which is the maximum 3D resolution recommended by the manufacturer. The suggested 3D resolution was derived from the scanner’s maximum 3D resolution of 0.2 millimetres. Outlier removal was performed by calculating the mean distance and standard deviation between neighbouring surface points. Surface points with a mean and standard deviation greater than an interval defined by the mean and standard deviation of all neighbourhood points were then classified as outliers and removed from the scene automatically. The 3D-noise level, which multiplies the standard deviation of neighbourhood points, controls outlier assignment. A higher 3D-noise level reduces identified outliers. Notably, our earlier manual point removal stage contributes to decreased noise on our surfaces. However, the specific process details remain undisclosed by the manufacturer, Artec3D.

Once both scans of the instrument were aligned and the background and noise were removed, fusion was applied to generate a watertight fusion mesh. A sharp and smooth fusion was performed, resulting in two STL models per scan. Sharp fusion contains a higher level of detail and achieves the maximum 3D resolution of 0.2 millimetres according to the manufacturer. The downside of this method is that potential noise left in the model after steps a-e from Fig. 4 may be intensified. The smooth fusion results in smoother models and despite the target resolution of 0.2 millimetres, the software may remove points to reach a maximum mean point distance of 0.6 millimetres. Additionally, models are automatically smoothed, and since surgical instruments are generally smooth, this might result in an aesthetically more appealing model. Unfortunately, the exact algorithms are not given by the manufacturer.

Fusion was performed with a resolution of 0.2 millimetres, ultra-HD sensitivity, and excluding frames above the maximum error threshold of 0.3 millimetres. In rare cases, the smooth fusion model was manually edited. These cases were the self-retaining retractors and speculums, where fine details and structures were not separated appropriately by the fusion process. In summary, while the sharp fusion models are always presented as is, the smooth fusion models are processed with the in-built smoothing function from Artec Studio, and manual editing in case of small structures which were over-smoothed by the software.

The models were inspected using the Artec Software and inside a rendering environment such as Blender. Eight instruments with movable parts, which may be present in different states in a surgical scene, were selected and scanned in different configurations. For example, some of the mouth gags were scanned with an open and closed state of the instrument. The final STL files are available in the data repository^30^.

### Data acquisition with autoscan inspec and ultrascan

Autoscan Inspec is a high-end industrial table desktop scanner ideal for reverse engineering of small parts and has become well known for its usage in dental applications. It utilises a blue light source and two five-megapixel grey-scale cameras. According to the manufacturer, its accuracy is 0.008 millimetre, resulting in an overall 3D resolution of 0.05 millimetres. Its max scanning area is 100 × 100 × 75 millimetres, although scanning the object in multiple orientations does allow for scanning larger objects.

The instrument was attached to the scanner’s robotic table at the end of its arm. The table can rotate 360 degrees and the arm itself can rotate from 0 to 135 degrees, with 50 being level with the desktop table. The scanner has a default path with 10 pre-settings of the table and arms rotational position, leading to 10 frames making up a single scan. If desired, the user can manually change these positions and the number of frames. There’s an “add scan” option, which allows users to scan extra frames in a desired position and add them to the earlier scan sequel. Only for a few thin, lengthy objects, a manual scan path was used. The default scan path, using a few additional scans upon visual inspection, was deemed sufficient for the remaining instruments. The scan was edited by removing unwanted data points, then, the instrument was rotated 180 degrees and scanned again. This process is called a flip scan. The two scans were either automatically aligned using the UltraScans proprietary alignment method, or semi-manually aligned if automatic alignment was not possible.

**Fig. 5:**
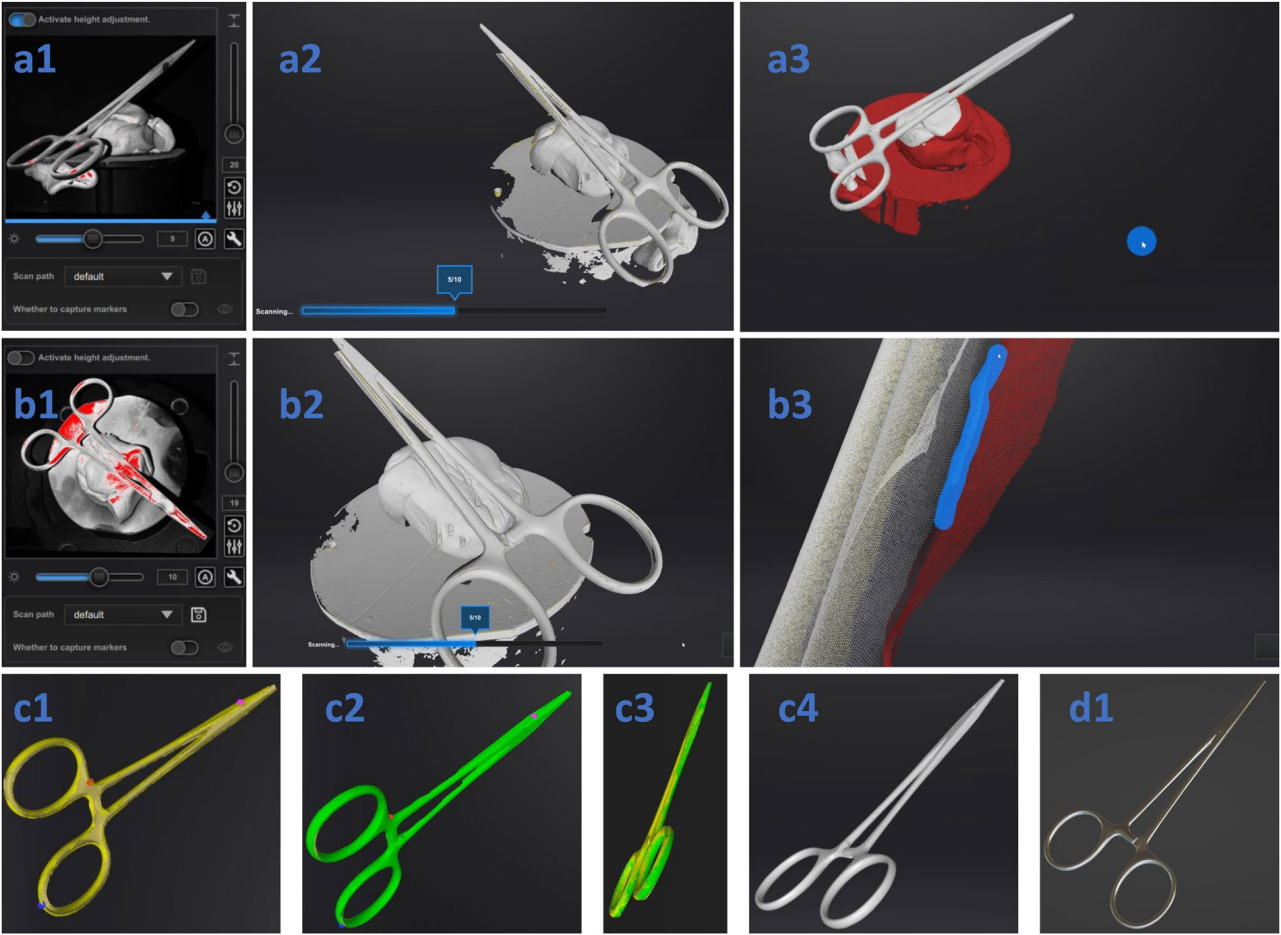
Data acquisition and processing workflow illustrated for the AutoScan Inspec with the UltraScan Software using Halsted Mosquito with tooth 1 as an example. Figure (a1 and b1) show the instrument sprayed with AESUB white. In (a2 and b2) an image of the scanning process is shown. (a3 and b3) displays manual editing of the scans. The steps shown in (b1-3) occur after (a1-3), when the instrument is manually turned in the scanner, also referred to as the flip scan. (c1-2) shows the semi-manual alignment procedure of (a) and (b), and (c3) the result. In (c4) final manual editing can be performed before exporting the result, shown in Blender with a metallic-like material (d1).

Automatic alignment identifies identical data points in both initial and flip scans from their respective point clouds and performs the alignment according to them. Given surgical instrument symmetry, automatic alignment is challenging and often fails. It was possible for less than 10 cases. For the remaining cases, semi-manual alignment had to be performed, introducing potential human error. Assessing alignment success is always performed by the post-processing operator. Figure 5c1,c2 shows semi-manual alignment, resulting in Figure 5c3. If automatic alignment succeeds, Figure 5c1,c2 steps were bypassed, directly displaying Figure 5c3. Undesired data in the point cloud post-alignment is then removed manually.

A watertight mesh was created from the resulting point cloud. Models were generated as STL twice: once with the ‘remove highlight’ function on, and once without. ‘Remove highlight’ eliminates spikes. Spikes are defined as triangles arising from point cloud data that deviate from the smooth surrounding surfaces. These spikes are often induced due to reflective surfaces. Unfortunately, the manufacturer, Shining 3D,does not provide exact algorithmic details for surface model generation.

Three instruments were scanned in different configurations. For example, we scanned a surgical knife in two configurations: one with the protection clip, and another with the protection clip removed.

Instruments not completely captured with two scans, the initial scan and the flip scan, were scanned with three or four scans instead. If the instrument did not fit into the scanner, or multiple additional scans overloaded the PC’s RAM, it was not scanned with the Autoscan Inspec, but the Artec Leo instead. The final STL files are available in the data repository^30^.

### Generating multiple likewise models

#### Blender add-on

**Fig. 6:**
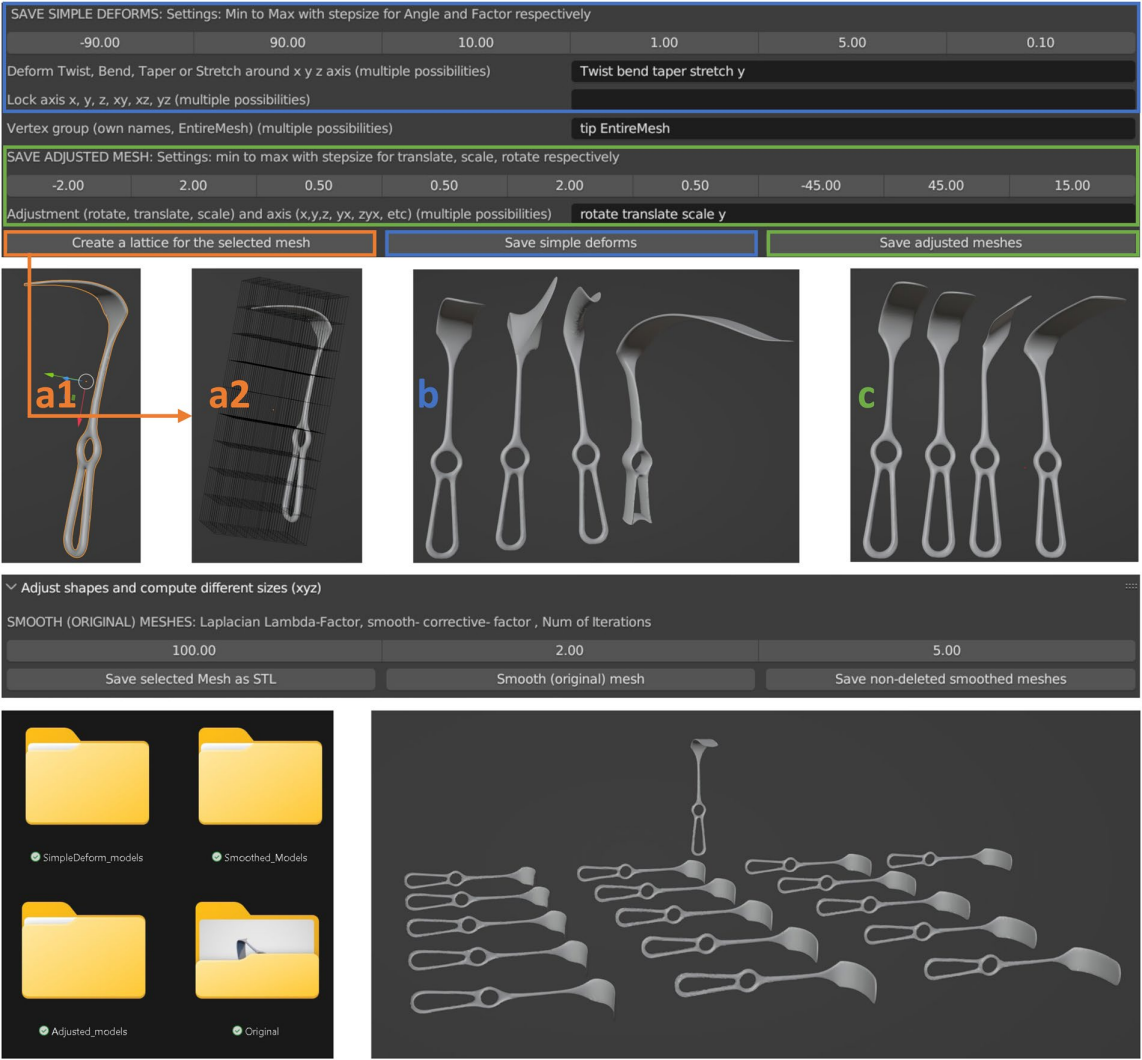
Example usage of the Blender Python add-on for creating likewise meshes based on a 3D scanned instrument. The Langenbeck Retractor Kochler Soft Tissue Wound Mikulicz was used to create this image. The lattice (a1-a2) is created at the top (in orange) and used to perform simple deforms (blue, b) and affine transformations, i.e., rotation, scaling or translation (green, c), on parts of the meshes. The left instrument in b and c is the original mesh, while the right side displays the mesh with various deformations and transformations made around the y-axis of the mesh group tip or the entire mesh. The user sets a minimum and maximum value for each desired model change, as well as a step size. The resulting models are automatically saved in folders depicted at the bottom left corner. The user can save the original model or create and save multiple varied smoothed models, shown at the bottom right corner, with the original model being the one with a different orientation.

To illustrate how virtual instruments can be easily transformed into multiple likewise instruments, we implemented a Blender Python add-on to assist in this task, see Fig. 6. The basis for the add-on are the simple deform function in Blender, allowing to bend, twist, taper and stretch the 3D meshes, and basic transformations, including rotation, translation and scaling.

Although these operations impact the entire model’s mesh, users have the option to automatically generate a fitting lattice, Figure 6a1-2, and manually define vertex groups within the lattice, such as those corresponding to handle and tip regions. A lattice is a 3D non-renderable deformation cage. When assigning these vertex groups within the lattice, they become accessible within the add-on. Predetermined groups can be linked to specific operations. This methodology ensures smoother transitions between vertex groups within the final mesh than working with the vertex groups directly.

The user can determine which operation to apply on which group of the lattice, including the minimum and maximum angle, amount of units or factors. A step size between the minimum and maximum value can be determined as well. After the user manually creates vertex group(s) and determines desired deform and transformation operation parameters, the add-on will automatically create all models and save them to the current Blender directory as STL files. It’s important to realise that the add-on will use the local axis of the model when specifying which action to perform around a certain axis.

If a factor or angle is zero, or scaling factor is one, the model is not saved since applying the change would not lead to a changed model. The same applies when rotation, scaling or translation operations are applied on the entire mesh. Even though the model’s position and orientation related to the world coordinate frame origin change, the model itself does not.

The add-on also has the option to create, show and save several differently smoothed models, using the subsurface, corrective smooth, Laplacian smooth and normal smooth modifiers. The add-on can also create multiple rescaled and smoothed meshes from all STL files found in a given directory path, e.g., the current Blender directory path. These functions have not been used in our case since we found that a Python script utilising the Trimesh library does this more efficiently and automatically, and it would also take enormous amounts of data storage. Therefore, we deemed it more appropriate to show an example and let potential users run the scripts themselves.

As an example of the Blender add-on usage, a single instrument from 12 different classes were modified using the Blender Python add-on. An overview of the settings and results while using the Blender add-on to create examples is given within the used instrument folder in the data repository^30^. The results for our example instruments can be found within the data repository^30^.

#### Python script

We furthermore provide a Python script to apply additional modifications to the instruments within our collection, to enlarge the dataset even further. Using the Trimesh library, this script has the capability of smoothing and scaling the meshes along their local axes. Suppose we scale one model with factors from 0.5 to 1.5 with steps of 0.1 along all three axes, we could generate 11^3^−1 = 1,330

likewise model. We subtract one likewise model since rescaling with a factor of one along all three axes results in an unchanged model. This can be useful as a form of data augmentation for creating deep-learning datasets. The Python script also implements automatic Taubin, Laplacian and Humpfrey smoothing on the input models^32,33^.

**Fig. 7:**
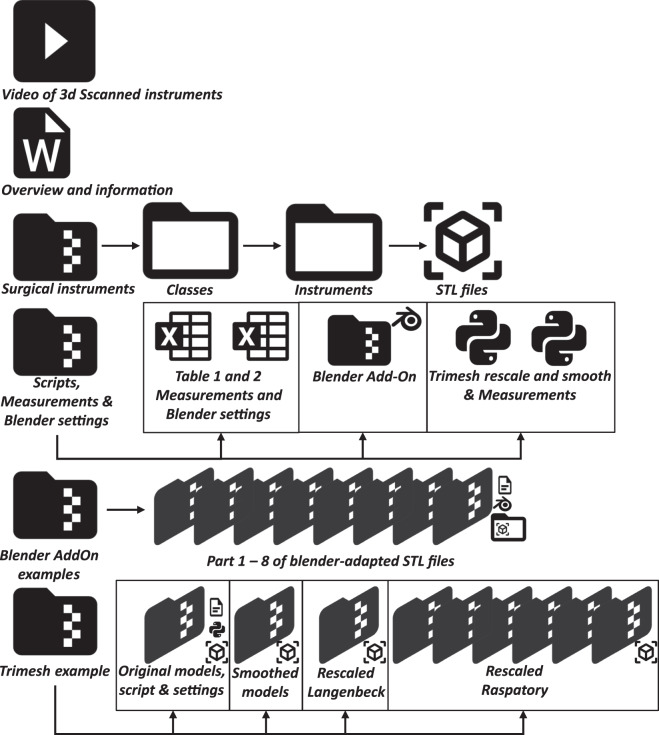
An overview of the contents within the data repository. It begins with a video showcasing the 3D-scanned instruments, showing one instrument per class. Thereafter a Word file with details on the number of models per class, which models were used for scanning in different configurations, and which are used for the Blender add-on and Trimesh examples. In a single folder all STL files resulting from the 3D scans are organized. After unpacking, the user can search per class and per instrument. The instrument folders contain STL files generated using the appropriate software, with the prefix “Leo” or “Inspec”, indicating the scanner used. A separate folder holds the measurements and the used blender settings for our examples in two separate Excel tables. This folder also includes the Python scripts for measurements, generating the reshaped 3D models and the Blender add-on. The example models made with either the Blender add-on or the Python script are in separate folders. Due to the size of the data, these are organized in multiple folders. Eight folders for the Blender example and nine folders for the rescaled and smoothed Trimesh example.

At the start of the process, the user is prompted to provide a main directory, along with inputs if scaling and smoothing are desired, along with the scaling factors and number of iterations, respectively. The script will then search for all STL files within the main directory and its sub-directories and apply the specified scaling and smoothing operations accordingly. The resulting files are exported to newly created folders, which mirror the folder structure of the main directory provided by the user. We provide examples for two instruments in our data repository^30^, as shown in Fig. 7.

#### Analysis

All scanned models were made watertight in the proprietary software and visually inspected in Microsoft 3D Viewer. The use of scanning spray and recommended settings as described in section “Instrument preparation” until “Data acquisition with Autoscan Inspec and UltraScan”, is expected to result in submillimetre precise scans, as specified by manufacturers Artec3D and Shining 3D.

Using Python with the Numpy and Trimesh libraries, all scanned models were aligned with a tight enclosing bounding box. The bounding box was automatically oriented to minimize its volume. From this bounding box, the width, height and length in millimetres were calculated. These measurements were compared to the physical model using a flexible millimetre ruler to test if the models were precise on a millimetre level. For this assessment, we utilised the virtual models without the “remove highlight” function (Autoscan Inspec) and models generated with sharp fusion (Artec Leo).

The Trimesh library was also used to calculate the volume of the models. When comparing models from identical scans, i.e., the smooth and sharp fusion, or the scans with and without removing highlights, the average difference is less than 1 millimetre in all directions.

We scanned *‘Arterial Clamp Halsted Mosquito 1’* twice with Autocan Inspec and *“Inspec Mouth Gag Denhart 1”* with both 3D scanners. The deviation between these virtual models was under 1.5 millimetres, suggesting that human error in the manual post-processing contributed less than 1.5 millimetre of error. Individual measurements can be found in “*Table 2 MeasurementsOnVirtualModelsUsingTrimeshLibrary”* in the data repository^30^.

## Data Records

The final data is stored in a repository^30^ according to the folder structure shown in Fig. 7. An overview of the classes is given in Fig. 2. The collection consists of scans of 103 surgical instruments. 49 instruments were scanned with the Artec Leo, resulting in double the number of STL files, one with sharp fusion settings and one with smooth fusion settings with minimal manual editing. 55 instruments were scanned with the Autoscan Inspec. For these scans, we provide one STL model created with the automatic “remove highlight” function, and one model without using this function.

We scanned 11 instruments in various configurations, including open and closed grip positions, as well as with and without blades on scalpel handles. Among these, eight instruments were scanned using the Artec Leo, and three instruments were scanned using the Autoscan Inspec. These specific instrument names can be accessed in the ‘Overview’ Word file available in the data repository^30^.

To demonstrate the capabilities of our provided add-on, we smoothed, deformed, or partially rotated, scaled, or translated 12 STL models from different instrument classes, resulting in 6,263 likewise instruments.

We furthermore applied our Python script, *‘RescaleAndSmooth_v2.py’* to create a multitude of similar models on two instrument classes, Chisels and Langenbeck Retractors, generating 5,380 STL by rescaling and smoothing.

For the original (non-deformed or scaled) 3D models, we report the total volume in cubic millimetres, length, width and height in millimetres. These measurements were performed on the virtual models using a Python script which is included in the data repository^30^.

We will also share these original instruments on MedShapeNet (https://medshapenet.ikim.nrw) to make them easily available and browsable by the medical imaging and computing community^31^.

## Technical Validation

The main issue regarding technical validation of the 3D models based on the medical instruments is the spatial geometry reconstructed by the scanners, Artec Leo and Autoscan Inspec, and their proprietary software. The manufacturers Artec 3D and Shining 3D evaluated the spatial 3D resolution to be 0.2 millimetre and 0.05 millimetre respectively. When taking into account that 3D scanning spray and recommended settings are used, submillimetre accuracy is expected.

Deviations in spatial geometry between 3D model and physical instrument are therefore likely due to manual post-processing. These may be due to unknowingly removing point cloud data while removing artefacts. They could also be caused by a misalignment of multiple scans while fusing them into a single model. Therefore, we manually compared the model’s length calculated using the Trimesh library with the instrument’s physical length. Still, since this measurement is difficult for three dimensional objects with curvatures, small errors might go unnoticed. We want to point out that these minor deviations won’t hamper the collections’ utility for our use cases mentioned throughout this article.

**Fig. 8:**
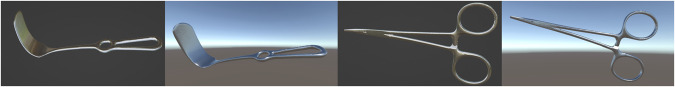
From left to right, a Langenbeck retractor and a needle holder scanned with Artec Leo visualised in Blender and Unity3D respectively.

A limiting factor of the presented data is the lack of real texture information, which is lost due to the necessary usage of 3D scanning spray. While transparent scanning sprays exist, we found that they significantly impede scanning accuracy. Therefore, we opted for precise scanning in lieu of real texture. The Autoscan Inspec is capable of scanning without scanning spray, but since less data is acquired without spray, producing a high-quality mesh requires too many flipscans, which makes post-processing infeasible. Realistic textures can, however, be applied easily using Blender or within a game engine, e.g., Unity3D (Unity Technologies, San Francisco, California, United States) or Unreal Engine, see Fig. 8 or Fig. 2.

Since we store our models in a standard format (STL), they are compatible with a large variety of visualisation and processing software.

## Usage Notes

STL is widely used for computer-aided design and manufacturing^34^. They can be analysed, manipulated and processed with open-source software such as Blender, 3D Viewer, Trimesh in Python and many more. The STL models could be used for computer vision applications such as instrument detection, segmentation, or tracking, as well as in medical mixed-reality simulation scenarios. To this end, the models can be integrated with game engines, such as Unity3D or Unreal Engine, to create mixed-reality apps or to render an unlimited number of photorealistic scenes. The models can directly be used or manipulated to have a more appropriate texture. With some adaptation, the models can also be used for 3D printing. The unprocessed, raw scan data, however, can only be accessed with the commercially available software of the proprietary 3D scanners and is of less interest for our mentioned use cases.

The material can be copied and redistributed in any medium or format. Furthermore, the data is free to adapt, remix, transform, and build upon the material. The data within this work is licensed under a Creative Commons Attribution 4.0 International License (CC BY 4.0) (https://creativecommons.org/licenses/by/4.0/).

## Data Availability

The proprietary software of Artec Leo and Autoscan Inspec was used for processing the scans. For the analysis of the dimensions of instruments, a Python script was written using the Trimesh, Numpy and Pandas library. Another Python script is provided for rescaling and smoothing the original models into a multifold of models, using deformations and affine transformations. All code is available in the data repository^30^.
